# A hypothesis on improving foreign accents by optimizing variability in vocal learning brain circuits

**DOI:** 10.3389/fnhum.2015.00606

**Published:** 2015-11-04

**Authors:** Anna J. Simmonds

**Affiliations:** Division of Brain Sciences, Computational, Cognitive and Clinical Neuroimaging Laboratory (C3NL), Imperial College LondonLondon, UK

**Keywords:** foreign accent, vocal learning, motor learning, non-native speech, language learning, variability, striatum

## Abstract

Rapid vocal motor learning is observed when acquiring a language in early childhood, or learning to speak another language later in life. Accurate pronunciation is one of the hardest things for late learners to master and they are almost always left with a non-native accent. Here, I propose a novel hypothesis that this accent could be improved by optimizing variability in vocal learning brain circuits during learning. Much of the neurobiology of human vocal motor learning has been inferred from studies on songbirds. Jarvis (2004) proposed the hypothesis that as in songbirds there are two pathways in humans: one for learning speech (the striatal vocal learning pathway), and one for production of previously learnt speech (the motor pathway). Learning new motor sequences necessary for accurate non-native pronunciation is challenging and I argue that in late learners of a foreign language the vocal learning pathway becomes inactive prematurely. The motor pathway is engaged once again and learners maintain their original native motor patterns for producing speech, resulting in speaking with a foreign accent. Further, I argue that variability in neural activity within vocal motor circuitry generates vocal variability that supports accurate non-native pronunciation. Recent theoretical and experimental work on motor learning suggests that variability in the motor movement is necessary for the development of expertise. I propose that there is little trial-by-trial variability when using the motor pathway. When using the vocal learning pathway variability gradually increases, reflecting an exploratory phase in which learners try out different ways of pronouncing words, before decreasing and stabilizing once the “best” performance has been identified. The hypothesis proposed here could be tested using behavioral interventions that optimize variability and engage the vocal learning pathway for longer, with the prediction that this would allow learners to develop new motor patterns that result in more native-like pronunciation.

## Introduction

### Vocal Learning

**Figure 1:**
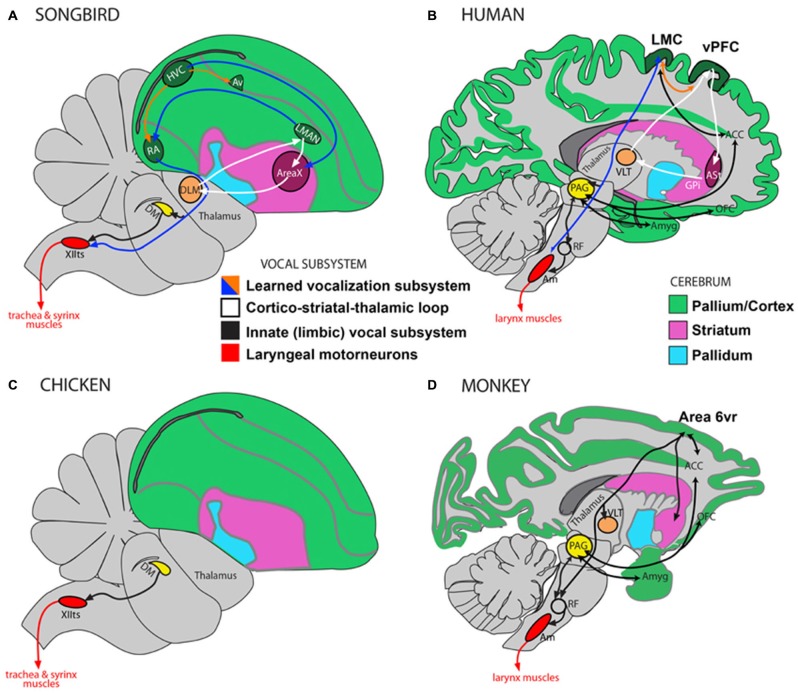
**Direct and indirect vocalization pathways in complex-vocal learners, limited-vocal learners and vocal non-learners.** Schematic of a songbird brain **(A)** and a human brain **(B)** showing the vocal motor pathway (blue arrow), the vocal learning pathway (white) and the laryngeal motorneurons (red). Also shown in **(B)** is the limbic vocal pathway for producing innate vocalizations (black). **(C)** Schematic of a vocal non-learning bird revealing the absence of forebrain song nuclei. **(D)** Schematic of limited-vocal learning monkeys showing presence of forebrain regions for innate vocalization and also of an indirect projection from a ventral premotor area (Area 6vr) to laryngeal motorneurons. Abbreviations: ACC, anterior cingulate cortex; Am, nucleus ambiguus; Amyg, amygdala; AT, anterior thalamus; Av, nucleus avalanche; DLM, dorsolateral nucleus of the medial thalamus; DM, dorsal medial nucleus of the midbrain; HVC, high vocal center; LMAN, lateral magnocellular nucleus of the anterior nidopallium; LMC, Laryngeal Motor Cortex; OFC, orbito-frontal cortex; PAG, periaqueductal gray; RA, robust nucleus of the of arcopallium; RF, reticular formation; vPFC, ventral prefrontal cortex; VLT, ventro-lateral division of thalamus; XIIts, bird twelfth nerve nucleus. Figure as originally published in Petkov and Jarvis (2012), reproduced with permission.

Vocal learning is the ability to imitate sounds that are heard, as opposed to producing innate vocalizations. Most mammals are not vocal learners and can only produce innate calls that remain unmodified throughout life (Petkov and Jarvis, 2012). Instead they are auditory learners and through experience can readily distinguish environmental sounds, making an appropriate response to what is heard, e.g., a command to “sit”, without the ability to produce it (Jarvis, 2004, 2006). In contrast, humans are highly skilled auditory and vocal learners. We are not born with speech and must learn by listening and practicing. Much of the neurobiology of vocal learning has been inferred from studies on songbirds and there are clear anatomical parallels between song learning birds and humans (Figure 1). Humans and songbirds both have a direct projection from motor cortex to motor neurons in the brainstem controlling movements required for vocalizations (larynx in humans and trachea and syrinx in songbirds). This projection is absent in non-learning birds such as chickens, and non-vocal learning primates, such as macaque monkeys (Petkov and Jarvis, 2012; Figure 1). Vocal learning, and motor learning more generally, involves the basal ganglia, which is the focus of the hypothesis presented here. It has been shown that basal ganglia circuitry is involved to a greater extent in motor learning than performance of acquired behaviors (Hikosaka et al., 1999, 2002). There have also been important distinctions made between different regions within the basal ganglia at different stages of motor learning, with the anterior striatum being involved in learning and the posterior striatum in production of overlearned automatic movements (Miyachi et al., 1997; Jueptner and Weiller, 1998; Graybiel, 2008; Yin et al., 2009). The hypothesis presented here focuses on the learning of foreign speech, which requires novel motor movements rather than previously acquired familiar articulatory movements used for native speech.

### Speech Acquisition in Infancy

Human infants begin speech acquisition by listening to speech in their environment. They are skilled both in auditory learning, memorizing the communicative sounds of people they interactive with, as well as in vocal learning, from babbling and single word production to articulating well-formed sentences. Stages of speech development start at a universal level and an infant has the ability to learn any language and will start learning the language to which they are exposed. At around 7 months for perception and 10 months for production, speech becomes language-specific. Although infants produce non-speech sounds from birth and vowel-like sounds at around 3 months, canonical babbling does not appear until around 7 months. Language-specific speech production is observed at around 10 months and word production at around a year (Kuhl, 2004; Simmonds et al., 2011b).

### Speech Acquisition Later in Life

In contrast, when older children and adults begin learning a foreign language, they do not start with a perception phase, a period of listening to language without attempting production of speech sounds. Instead they begin producing speech early on in the learning process, at the same time as undergoing auditory learning. Unlike infants, older learners do not undergo a babbling phase but move straight to word meaning and phrase production, which is influenced by the native language. Using a listening task in bilinguals who learnt a second language after the age of 12, it has been shown that there is a strong tendency to translate a word in a foreign language (L2) into its native (L1) equivalent (Thierry and Wu, 2007; Wu and Thierry, 2010). Similarly, during L2 covert word production, both L2 and L1 phonological representations are retrieved (Wu and Thierry, 2011). Proficient use of vocabulary and grammar are essential skills, but can be learnt instructively, for example from books. However, acquiring a native-like accent requires repeated motor practice, with the accuracy of articulation dependent on repeated attempts to match auditory exemplars of correct pronunciation. Even then, there is considerable inter-individual variability in achieving accurate pronunciation, both in terms of learning strategies and in attainment (Bley-Vroman, 1990) and individual differences in performance have been shown to correlate with structural brain differences (Golestani and Pallier, 2007; Golestani et al., 2007). The challenge of speaking a foreign language is a problem faced by students and teachers of second language education around the world, and pronunciation errors substantially affect communication skills.

This challenge has effects on both the spoken performance in a foreign language, and the neural systems involved. The “native-likeness” of an accent, as judged by native speakers, declines over time as the age at which the speaker starts using the foreign language increases. Italian immigrants arriving in the US were deemed to have a native-like accent if they arrived before the age of two, whereas those arriving as teenagers or young adults had accents that clearly marked them as non-native speakers (Flege, 1995). Perhaps one of the most famous examples of a marked foreign accent in a highly proficient user of a foreign language is Józef Teodor Konrad Korzeniowski, better known by his anglicized name, Joseph Conrad. As a late learner of English as a foreign language he mastered the language to such an extent that he was able to produce great works of fiction in English (his *third* language), yet was left with such a thick Polish accent that he was reported to be incomprehensible. Scovel (1988) coined the term the “Joseph Conrad phenomenon”, referring to the mismatch between lexical, morphological and syntactic proficiency, and pronunciation. Even for highly proficient bilinguals, having learnt a language later in life results in differences in activation patterns during speech production. Speaking in a non-native, relative to native, language requires greater engagement of motor-sensory control systems (Simmonds et al., 2011a).

In addition to age at the time of learning, other factors claimed to affect the degree of foreign accent include gender, amount of time spent in an L2-speaking environment, amount of L1 and L2 use, formal instruction, motivation and language learning aptitude (Piske et al., 2001). Another explanation for the failure to acquire the native accent in a foreign language is that late bilinguals use the same syllable representation for both of their languages, which results in producing non-native L1-like patterns in their L2. In contrast, early bilinguals have separate representations for their two languages, even for syllables that are shared across the languages (Alario et al., 2010). The present article presents a novel hypothesis on what might explain the persistent accent in late language learners and considers how it could be improved. The hypothesis is informed by findings from vocal learning research in songbirds and motor learning more generally, as well as our previous work particularly focusing on the response of the anterior striatum during adult human vocal learning (Simmonds et al., 2014). Although the anterior striatum was initially active during production of unfamiliar foreign speech, activity in this region rapidly declined. The decline in the striatum happened over the course of the first scanning session, even before formal training. No decline was found for pronunciation of native non-word stimuli, indicating that the reduction was not an effect of novelty. These findings suggest that late language learners do not maintain use of the vocal learning pathway during learning. Although no direct comparison has been made between early and late language learners in terms of activity in the basal ganglia-forebrain-thalamic circuit, a likely finding would be that early learning of a native language would engage this circuit. However, without research on human infants during speech acquisition, this remains speculative.

### Parallels Between Song Learning Birds and Humans for Song and Speech

As discussed above, humans are highly skilled auditory and vocal learners. Vocal learning also exists in parrots and oscine songbirds (order: Passeriformes; Mooney, 2009; Petkov and Jarvis, 2012), hummingbirds (Jarvis et al., 2000), and to a far lesser degree, some of the traits associated with vocal learning also exist in mice (Arriaga and Jarvis, 2013). The hypothesis presented here is grounded in findings from the avian literature on song learning. There are a number of neural and behavioral parallels between humans and songbirds (see Doupe and Kuhl, 1999; Mooney, 2009; Fee and Goldberg, 2011; Sakata and Vehrencamp, 2012; Brainard and Doupe, 2013; Bertram et al., 2014; Woolley and Kao, 2015). In the same way as human infants learning speech, songbirds also begin vocal learning with a perception phase, during which they listen to songs from a tutor (Doupe and Kuhl, 1999; Brainard and Doupe, 2000; Konishi, 2004). Without exposure to adult song, production of accurate vocalizations is not possible. The production phase in songbirds begins with “subsong”, (similar to human babbling), before moving onto “plastic song” (while they practice what they are learning), before “crystallized” song (the equivalent of human native speech) appears. During the plastic song stage, songbirds use trial-and-error learning to adjust their vocal performance until the auditory feedback from their vocal output matches the auditory templates acquired during the auditory learning phase (Brainard and Doupe, 2000; Mooney, 2009; Bolhuis et al., 2010).

As well as similarities in the developmental progression of learning, human speech learning and birdsong acquisition have parallels at the neural and genetic levels (Jarvis et al., 2005; Ölveczky et al., 2005; Bolhuis et al., 2010; Pfenning et al., 2014). A recent gene expression study examined transcriptional specializations in humans and song-learning birds and found that the songbird RA nucleus is most similar to layer 5 neurons of human laryngeal motor cortex (LMC; Pfenning et al., 2014). The songbird Area X in the striatum is most similar to a region within the human anterior striatum (Pfenning et al., 2014), and data from our recent vocal learning study on humans support this finding (Simmonds et al., 2014). The songbird HVC is similar to layers 2 and 3 neurons of primary motor cortex, and thereby possibly also to LMC; songbird LMAN has a weak similarity to Broca’s area that requires further investigation for confirmation; DLM (dorsolateral nucleus of the medial thalamus) is most similar to the human anterior thalamus necessary for speech learning and production (Jarvis, 2004; Petkov and Jarvis, 2012).

In this article I present a hypothesis on how foreign accents could be improved by optimizing variability in vocal learning brain circuits, followed by support for the hypothesis, drawing on the literature on variability in songbird vocal learning and variability in motor learning. The article concludes with approaches for testing the hypothesis.

## Hypothesis (Figure 2)

The hypothesis presented here is that, as songbirds do, humans have a vocal learning pathway that controls neural and behavioral variability and the influence of this pathway is reduced in older learners, which leads to an inability to master the native accent when learning new languages. Furthermore, if this variability can be optimized in late learners, vocal learning could perhaps be more complete and thereby reduce or eliminate the foreign accent. The focus here is on variability in the acoustic structure of speech, rather than sequencing or timing variability.

### Hypothesis Part 1: The Vocal Learning Pathway in Late Language Learners Becomes Inactive Too Early in the Learning Process and Prevents Accurate Pronunciation in a Foreign Language

In 2004, Erich Jarvis (Jarvis, 2004, 2007) put forward the hypothesis that as in songbirds there exist two pathways in humans: one for vocal learning, and one for production of previously learnt speech. Learning novel motor sequences that are necessary for accurately pronouncing foreign speech is a challenge, and in this article, I argue that for late learners of a foreign language, the vocal learning pathway becomes inactive too early in the learning process, engaging the motor pathway once again. Consequently these late learners do not acquire novel sequences of articulatory movements for the new speech; instead they adapt existing production sequences, which results in speaking the new language with an accent influenced by their own first language, rather than mastering the native-like accent of the target language.

**Figure 2:**
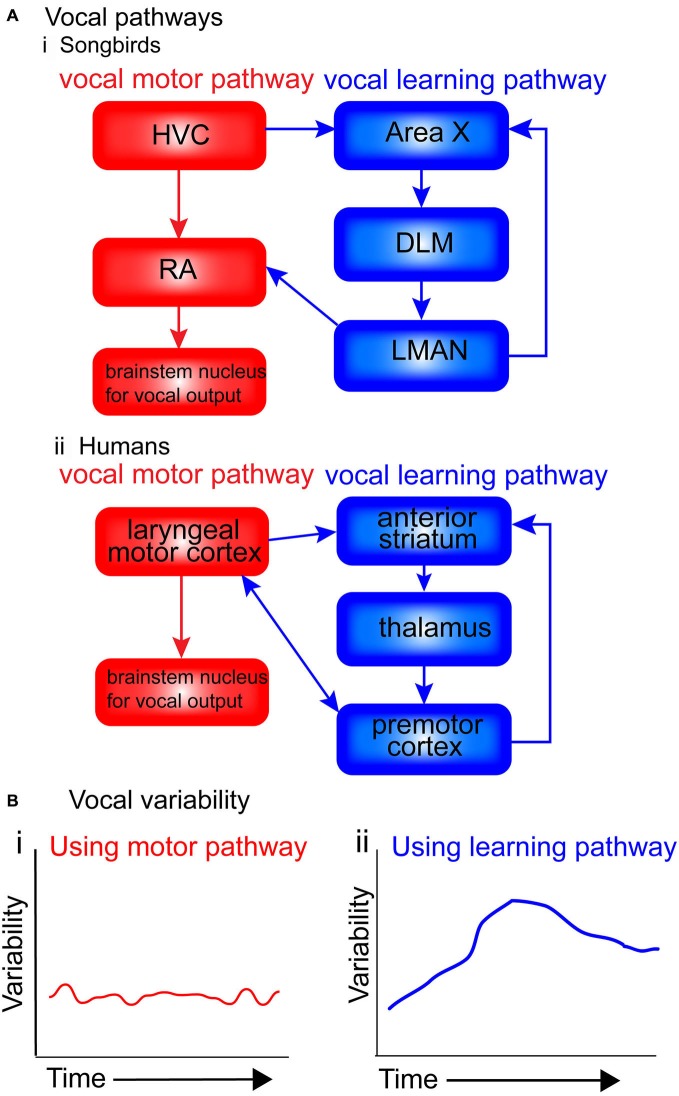
**Motor and vocal learning pathways in songbirds and humans and the role of variability. (A)** simplified diagram of the pathways involved in vocal learning and production in songbirds and humans. **(i)** In songbirds, the vocal motor pathway used for production of established song [shown in red: HVC, RA (robust nucleus of the arcopallium) and brainstem nucleus for vocal output] is used to produce the song. The vocal learning pathway [AFP: anterior forebrain pathway, shown in blue: Area X, dorsolateral nucleus of the medial thalamus (DLM) and LMAN (lateral magnocellular nucleus of the anterior nidopallium)] is used in songbirds during the acquisition of the pattern in song learning. **(ii)** In humans, the motor pathway (shown in red: laryngeal motor cortex and brainstem nucleus for vocal output), and the vocal learning pathway (shown in blue: anterior striatum, thalamus and premotor cortex). **(B)** Suggested levels of vocal variability when using the two pathways. I suggest that when using the motor pathway **(i)**, production is stable, with little trial-by-trial variability. When using the vocal learning pathway **(ii)**, trial-by-trial variability gradually increases, reflecting an exploratory phase in which the learners try out different ways of pronouncing the words (‘motor exploration’), before decreasing and stabilizing once the ‘best’ performance has been identified (‘motor exploitation’).

Figure 2A presents a simplified diagram of the motor and vocal learning pathways in songbirds and humans. In both songbird pathways the HVC ultimately projects to motor neurons in the brainstem (the nXllts), which then projects to the vocal muscles for vocalization. Following the vocal motor pathway, the HVC projects directly to the RA, which in turn makes a direct projection to brainstem vocal motor neurons (see Figure 2A_i_. The vocal learning pathway (anterior forebrain pathway—AFP) consists of a cortical-basal-ganglia-thalamic loop similar to mammals, involving Area X, the DLM and LMAN (Jarvis, 2004, 2006). This loop can be further segregated into lateral and medial loops, both receiving input from HVC into Area X, but with different outputs. The output of the lateral loop is from LMAN to RA; the output of the medial loop is from MMAN (medial magnocellular nucleus of the midbrain) to HVC (Jarvis, 2006). The HVC continues developing until month four post-hatch, near the end of the plastic-song stage (Alvarez-Buylla et al., 1992).

In songbirds the vocal learning pathway is involved during the acquisition of the song pattern and remains important for the modulation of song across social contexts. The vocal motor pathway is involved in producing the learned song (Nottebohm, 2005), and during the plastic song stage in juveniles both pathways interact (Ölveczky et al., 2005). Subsong in juvenile birds does not require HVC, a key premotor area for singing in adult birds, but does require activity in RA and LMAN, which is involved in learning but is not necessary for adult singing of an established song (Aronov et al., 2008). Therefore the relative contributions of the vocal motor and learning pathways seem to change across development in songbirds. It is likely that a similar shift in balance between the two pathways occurs in humans at different stages of learning. I suggest that in late learners of a foreign language, the vocal learning pathway is involved to a greater extent at the beginning of the learning phase but before learning is complete, the balance in activity between the two circuits shifts more to the motor pathway once again, which prevents accurate learning of pronunciation.

“Closed-ended learners”, such as the zebra finch, are unable to learn a new song in adulthood, even with an intact AFP (Brainard and Doupe, 2002; Funabiki and Funabiki, 2009), as the song they learn becomes crystallized at around 90 days post-hatch and remains stable throughout adulthood (Brainard and Doupe, 2002). An “open-ended learner”, such as a canary, is able to repeat the learning process in adulthood (Nottebohm et al., 1976; Brainard and Doupe, 2002). If a region within the vocal learning pathway is lesioned in an adult open-ended learner, the bird can continue to produce song it had previously learnt, but is unable to learn a new song (Brainard and Doupe, 2000, 2002; Brainard, 2004). In humans, subcortical structures including the basal ganglia, similar to regions within the songbird AFP, modulate production of overlearned language (e.g., poems or quotations), automatic speech (e.g., counting or reciting the days of the week) and formulaic expressions or fillers (Bridges et al., 2013). Patients with lesions in these regions produce fewer examples of formulaic language than controls (Sidtis et al., 2009). This suggests that overlearned language relies more on subcortical structures than novel language does, perhaps reflecting less reliance on the vocal learning pathway in later language learning.

### Hypothesis Part 2: Variability in Neural Activity Within Vocal Motor Circuitry Generates Vocal Variability that Supports the Acquisition of Native-Like Pronunciation in a Foreign Language

Further, I suggest that prolonged random variation is an essential prerequisite for vocal learning, and optimal variability within the vocal learning pathway generates vocal variability and supports accurate pronunciation with a native-like accent. Activity within the vocal learning pathway in adult songbirds remains important for real-time generation of spectral variability necessary for adapting the song based on different social contexts. In songbirds, vocal variability is actively injected into the premotor song-control region RA (robust nucleus of the arcopallium) by the LMAN (lateral magnocellular nucleus of the anterior nidopallium), which is the output of the vocal learning pathway (Goldberg and Fee, 2011). The LMAN is not necessary for the production of song, only learning and modification to it. When LMAN neurons are inactive, the vocal motor pathway produces an accurate, established pattern. When the LMAN is active during song production, there is much more variability in the song. This variability is needed to reach accurate imitation of a pattern. I argue that in humans, strategies that increase the variability of neural activity in the vocal learning pathway may increase behavioral variability and exploration and promote more successful learning.

Figure 2B presents suggested levels of vocal variability when using the two pathways. I suggest that when using the motor pathway, production is stable, with little trial-by-trial variability. When using the vocal learning pathway, trial-by-trial variability gradually increases, reflecting an exploratory phase in which the learners try out different ways of pronouncing the words, before decreasing and stabilizing once the “best” performance has been identified.

## Support for the Hypothesis

In this article I argue that if variability can be optimized in late language learners, vocal learning could perhaps be more complete and thereby enable mastery of a native-like accent in the foreign language. It is not simply that variability in vocal learning needs to increase. Too much variability, or noise, prevents learning just as too little does (Faisal et al., 2008). Therefore, for effective learning it is necessary to optimize the amount of variability. By trying different versions of producing the target, a learner is able to monitor outcomes and refine the movement sequences that result in the most desired outcome. This is true in songbirds and is likely true in humans as well.

### Variability in Songbird Vocal Learning

A critical amount of noise within the song production pathway is necessary during song learning (Doya and Sejnowski, 1995; Ölveczky et al., 2005) and song variability is generated by the AFP (Woolley and Kao, 2015). This variability in the AFP has been shown to correlate with performance variability (Kao et al., 2005; Woolley and Kao, 2015). Although a critical amount of noise appears essential for songbird learning, optimal learning will only occur within the appropriate level of noise for a given stage of learning. There is a reduction in variability within the AFP as the song crystalizes, although some neural and vocal stochastic variability is present even in adult songbirds with apparently stable song (Kao et al., 2005; Kao and Brainard, 2006; Andalman and Fee, 2009). Using altered auditory feedback in adult songbirds, Tumer and Brainard observed that birds were able to learn how their song changed as a result of small variations in vocal performance (Tumer and Brainard, 2007). They suggest that residual variability that persists in well-learned skills reflects motor exploration as part of the trial-and-error learning and monitoring processes, and that this helps to support continuous learning and optimization of performance.

Within the AFP song learning pathway, lesions to Area X have little or no effect on song variability during the vocal babbling stage (Goldberg and Fee, 2011), but when Area X is lesioned in juveniles, the song does not fully crystallize as they become adults and instead remains variable (Sohrabji et al., 1990; Scharff and Nottebohm, 1991). In contrast, LMAN inactivation results in reduced, almost absent variability in song in juveniles and adults (Kao et al., 2005; Ölveczky et al., 2005; Kao and Brainard, 2006; Aronov et al., 2008; Thompson et al., 2011). Young birds at an early stage of song development, which have the most variable song performance, show the greatest reduction in song variability following LMAN inactivation (Ölveczky et al., 2005). Similarly, during vocal babbling in juveniles a lesion to the DLM, part of the thalamus that receives output from the basal ganglia, almost completely removes variability and causes the birds to produce a stable stereotyped song (Goldberg and Fee, 2011).

A decrease in variability has also been observed following lesions to the dorsal arcopallium, adjacent to RA, by authors who suggest this to be an auditory region involved in song learning (Bottjer and Altenau, 2010). However, this region, along with other brain areas adjacent to the vocal systems of vocal learning birds, has been shown to be active during limb and body movements (Feenders et al., 2008). This suggests that the systems involved in vocalizations are controlled by a cerebral motor system. Although a similar auditory pathway exists in both vocal learners and non-learners, vocal learners have a specialized vocal motor system that enables auditory input to be translated into vocal signals (Feenders et al., 2008). A recent electrophysiology and lesion study supports this motor hypothesis, again showing motor behavior and movement control of this region (Mandelblat-Cerf et al., 2014). Further support for the motor hypothesis comes from Pfenning et al. (2014) who, using gene expression, found that the molecular profile of this region is similar to that of the motor and premotor cortex in primates, and not the auditory cortex. Therefore, the variability observed by Bottjer and Altenau (2010) may be similar to that found in RA and motor pathways.

In trial-and-error learning in juvenile songbirds the “trial” is represented by the variability in the song, reflecting the motor exploration phase, and the “error” is represented by evaluation of song performance, based on auditory feedback (Tumer and Brainard, 2007; Andalman and Fee, 2009; Sober and Brainard, 2009; Fee and Goldberg, 2011). Such variability is necessary for reinforcement-based trial-and-error learning, as the learning process requires exploration of a range of action sequences, evaluation of performance with each and modifications to behavior that result in improved performance (Ölveczky et al., 2005).

Even in crystallized song in adult birds, trial-by-trial variability persists. This variability supports ongoing motor exploration, which maintains performance and makes modifications when necessary (Tumer and Brainard, 2007). Song variability is also context-dependent. During “directed” song, in which a male sings a courtship song to a female, the sequencing and structure of syllables are much less variable than when the male sings alone (“undirected” song; Kao et al., 2005; Ölveczky et al., 2005; Kao and Brainard, 2006; Teramitsu and White, 2006; Sakata et al., 2008; Kojima and Doupe, 2011; Woolley et al., 2014). This suggests that singing alone reflects a practice state of exploratory vocal learning, and directed singing reflects a performance state, in which the male produces the best rendition of their song they memorized during the sensitive period in development (Kojima and Doupe, 2011). LMAN activity is much greater and more variable during undirected song than during directed song (Hessler and Doupe, 1999; Kao et al., 2005; Kao and Brainard, 2006; Kojima and Doupe, 2011; Brainard and Doupe, 2013; Woolley and Kao, 2015) and a lesion to LMAN removes the variability and causes undirected singing to be much more consistent (Kao et al., 2005; Kao and Brainard, 2006; Hampton et al., 2009).

### Variability in Motor Learning

The hypothesis proposed here is also supported by findings from research on motor learning more generally. Noise in general motor learning (not just vocal learning) has been defined as a mismatch between expected and actual sensory feedback that is not necessarily related to performance errors (Faisal et al., 2008). Recent theoretical and experimental work suggests an important role for noise, termed stochastic facilitation, in motor learning, i.e., variability or noise in the motor movement is necessary for the development of expertise (McDonnell and Ward, 2011; Mendez-Balbuena et al., 2012). Stochastic processes, introducing variability in the execution of motor movements, permit a full exploration of the learning space. Motor learning involves an “exploration” phase, during which trial-and-error learning is performed to identify the optimal movement for a successful outcome. Once that is identified, the learner moves into the “exploitation” phase, in which they continue producing that movement until the necessary outcome is achieved. Motor learning therefore involves a tradeoff between performing multiple movements to find the one that most reliably produces the desired outcome, and continuing to produce that movement once it has been identified (Müller and Sternad, 2009; Ravbar et al., 2012). During the exploration phase performance is highly variable, and it becomes more consistent when the average performance is closer to the target outcome, suggesting that variance decreases with the bias (Müller and Sternad, 2009; Ravbar et al., 2012). The tradeoff between exploration and stabilization is not the same throughout the learning process. When learning continuous actions (such as dancing), different components of the action may need exploratory variability while others, which may be closer to the target, require stabilization (Doya, 2000). With this type of approach, breaking the movements down into segments would allow variability to be regulated locally so that only those parts of the action that need to change the most undergo exploration, i.e., learning based on the local bias (Doya, 2000; Ravbar et al., 2012).

Individual differences in the amount of motor variability have been associated with the ability to learn or adapt motor skills (Sober and Brainard, 2012; Wu et al., 2014) and models of trial-and-error learning suggest that previous performance can predict the amount of variability in the motor output (Kao et al., 2008). This suggests that motor “noise”, or variability, is a central component of motor learning (Herzfeld and Shadmehr, 2014). Neural variability is also an indicator of motor learning. As motor habits form, spike firing in the ventromedial striatum peaks at the beginning and end of the motor sequence, and changes to this firing have been suggested to be a sign of learning (Howe et al., 2011). In non-vocal motor learning in rodents, using a reward-based conditional T-maze task, spiking of striatal neurons has been shown to be highly variable at the initial stage of learning, but following training became more consistent (Barnes et al., 2005). The variable firing rate during learning is considered to represent “neural exploration”, whereas the stable firing after learning reflects “neural exploitation”.

## Testing the Hypothesis

This converging literature from research on songbird vocal learning and more general motor learning motivated our previous work, which suggests that in late learners of a second language, the vocal learning pathway may become inactive too early, ending the motor learning phase prematurely. Instead, the motor pathway is recruited once more, which results in the learner producing the original native motor patterns for speech; this results in speaking with a foreign accent (Simmonds et al., 2014). The hypothesis proposed here could be tested using behavioral interventions that keep speakers in the learning phase (engaging the vocal learning pathway) for longer, with the prediction that this would allow them to develop new motor patterns that result in more native-like accuracy of pronunciation. This could be investigated using strategies that induce neural and behavioral variability, such as altering the auditory feedback that learners receive. Disrupting auditory feedback in songbirds results in rapid changes to learned song (Tumer and Brainard, 2007; Andalman and Fee, 2009; Hoffmann and Sober, 2014), although variability itself did not increase in these studies. This suggests that altering auditory feedback induces experimentally controlled “errors” and changes in song performance (Tumer and Brainard, 2007; Andalman and Fee, 2009; Fee and Goldberg, 2011). Sakata and Brainard (2008) have also found populations of neurons that appear to be sensitive to auditory feedback. Dramatic changes to auditory feedback can increase song variability and decrystallize the song (Leonardo and Konishi, 1999). Using Bengalese finches, Woolley and Rubel have demonstrated that temporary deafening leads to the rapid deterioration of syllable structure and an increase in vocal variability, but once hearing is restored, song is produced normally again (Woolley and Rubel, 1997, 2002). Therefore, although altered auditory feedback disrupts speech production, the auditory template of the acoustic template could remain intact. Assessing speech perception as well as production would identify whether the motor pattern or auditory target has been impaired.

Altered auditory feedback has also been shown to affect vocal production in humans (Houde and Jordan, 1998; Jones and Munhall, 2005; Tourville et al., 2008; Lametti et al., 2012; Kort et al., 2014; Ogane and Honda, 2014), although its role in language learning has not been explored. Different types of feedback could be used to investigate different ways of modulating variability during vocal learning, manipulating cognitive and motor processes to promote variability. Types of auditory feedback could include frequency-altered, delayed, background noise or white noise. Behavioral variability could be assessed by analyzing the acoustic properties of participants’ speech, including simple measures of intensity, duration and frequency, as well as correlations of the long-term spectra of specific words and characterization of formants. Somatosensory feedback could also be manipulated, for example altering jaw movements during speech, which has been shown to result in a mismatch between the expected sensations and the sensory feedback actually received, which causes somatosensory error signals that lead to compensatory movements (Tourville et al., 2005; Guenther et al., 2006). Some speakers rely on auditory feedback information and others rely more on somatosensory feedback (Lametti et al., 2012). Investigating a range of alterations to feedback would allow optimization of variability.

Using continuous speech at the sentence level would allow evaluation of performance to be carried out locally, focusing on specific words or phonemes. Rather than aiming to adapt a speaker’s overall level of variability, altered feedback could be used to only induce motor exploration in sounds that need to change. By assessing an individual’s speech, feedback manipulations could be developed to only occur for certain words. This type of approach has previously been investigated in zebra finches by manipulating song learning so that only a specific part of the song requires vocal exploration. Ravbar et al. (2012) found no apparent increase in the variability of one syllable when a second first appeared, demonstrating that the bird was able to rapidly switch between performing a highly stereotyped and a highly variable syllable.

The hypothesis proposed here could also be tested using neurobiologically-plausible computational simulations of the neural systems involved in vocal learning. The known neuroanatomy and structural connections of networks involved in speech production, defined using imaging studies, could be used to create a neuroanatomically-constrained model to simulate behavioral variability and learning effects. This type of model would help explain how neural and behavioral stochastic facilitation, with a focus on the striatum as a mediator, could affect vocal learning and allow us to explore, theoretically, the most effective amount of stochastic variability for successful learning. This would also allow for theoretical investigation of the influence of stochastic processes on learning and to simulate interventions in order to predict the optimal level of induced variability for best learning. Larger projects could then investigate the long-term benefits of these novel strategies for foreign language learning, which could lead to the development of new training materials with a strong evidence base, and discussions with educational policy-makers directing future strategies for improving foreign language learning outcomes.

## Funding

This work was supported by The Leverhulme Trust, award number RPG-2013-258.

## Conflict of Interest Statement

The author declares that the research was conducted in the absence of any commercial or financial relationships that could be construed as a potential conflict of interest.
